# Bilateral Pleural Effusions as an Initial Presentation in Primary Sjögren's Syndrome

**DOI:** 10.1155/2012/640353

**Published:** 2012-11-01

**Authors:** Go Makimoto, Michiko Asano, Nobukazu Fujimoto, Yasuko Fuchimoto, Katsuichiro Ono, Shinji Ozaki, Koji Taguchi, Takumi Kishimoto

**Affiliations:** ^1^Department of Respiratory Medicine, Okayama Rosai Hospital, 1-10-25 Chikkomidorimachi, Minami-ku, Okayama 7028055, Japan; ^2^Department of Respiratory Medicine, National Hospital Organization Okayama Medical Center, 1711-1 Tamasu, Kita-ku, Okayama 7011192, Japan; ^3^Department of Pathology, Okayama Rosai Hospital, 1-10-25 Chikkomidorimachi, Minami-ku, Okayama 7028055, Japan

## Abstract

Sjögren's syndrome (SS) is a systemic autoimmune disease characterized by sicca symptoms. Interstitial pulmonary fibrosis and tracheobronchial sicca are the most common symptoms of pulmonary involvement in primary SjS, and they are rarely accompanied by serositis such as pleuritis or pericarditis. We report a case of SS presenting initially with bilateral pleural effusions. A 63-year old man was admitted to our hospital with a one-month history of cough, dyspnea, and right chest pain. Chest-computed tomography revealed bilateral pleural effusions. Serum anti-SS-A antibody titer was 1 : 256. Ophthalmological examination revealed a positive Schirmer test. Lip biopsy showed atrophy and plasmacytic infiltration of the salivary gland. Corticosteroid treatment was initiated. Pleural effusions were almost completely resolved by day 30. The patient has not experienced any recurrence.

## 1. Introduction

Sjögren's syndrome (SS) is a systemic autoimmune disease characterized by sicca symptoms. Pathologically, chronic inflammation is seen in the lacrimal glands and small salivary glands. Interstitial pulmonary fibrosis and tracheobronchial sicca are the most common symptoms of pulmonary involvement in primary SjS, and some cases are also complicated by pulmonary arterial hypertension, pseudolymphoma, pulmonary lymphoma, lymphocytic interstitial pneumonitis, and amyloidosis [1, 2]. However, they are rarely accompanied by serositis such as pleuritis or pericarditis. We report a case of SS presenting initially with bilateral pleural effusions (Table 2).

## 2. Case Report

 A 63-year-old man was admitted to our hospital in December 2011. He had a history of diabetes mellitus, prostate enlargement, and brain infarction. He reported being in his usual state of health until approximately one month earlier, when he developed cough, dyspnea, and right chest pain. He went to a local clinic where computed tomography (CT) of the chest revealed bilateral pleural effusions.

**Figure 1:**
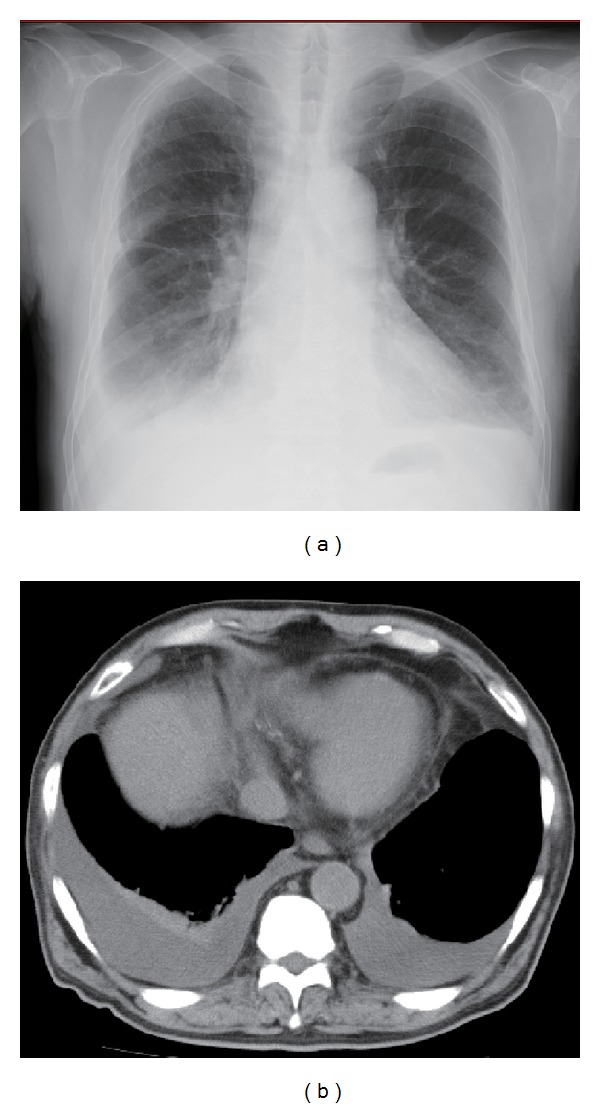
Chest X-ray (a) and computed tomography (b) showed bilateral pleural effusion without any consolidation or ground glass opacities in the lung.

 The patient had no fever, rash, joint swelling, or pain. Chest X-ray (Figure 1(a)) and CT (Figure 1(b)) showed bilateral pleural effusions. Laboratory findings on admission were as follows: white blood cells (WBCs) 5700/*μ*L, C-reactive protein (CRP) 9.7 mg/dL, and erythrocyte sedimentation rate (ESR) 100 mm/hr. In addition, the patient was found to be hypothyroid: free thyroxine 4 was 0.59 ng/dL, thyroid stimulating hormone was 48.71 *μ*IU/mL, and antithyroglobulin antibody was over 4000 IU/mL. Pleurocentesis revealed an exudative pleural fluid with no malignant cells but increased proportion of lymphocytes. Protein/albumin concentration and lactase dehydrogenase value in the fluid were 5.6/2.3 g/dL and 315 IU/L, respectively. The smear tests for *Mycobacterium tuberculosis* and bacterial culture were negative.

**Figure 2:**
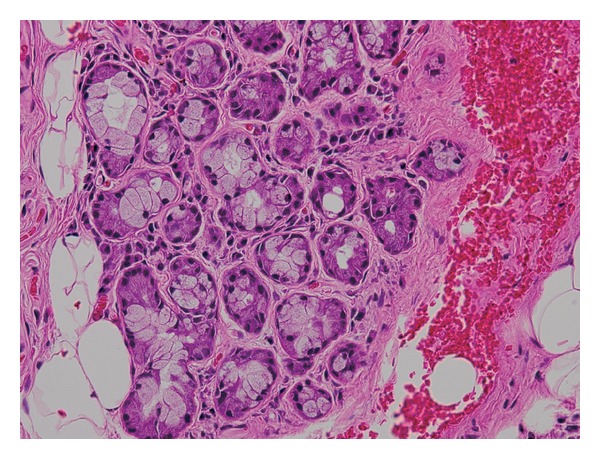
Lip biopsy showed atrophy of the salivary gland and plasmacytic infiltration around the salivary gland ducts.

 Antibiotic therapy (tazobactam/piperacillin 4.5 g × 3/day) was initiated and serum CRP decreased to 2.8 mg/dL on the third hospital day. However, the patient developed high fever on the fifth day. Laboratory evaluation for lupus erythematosus was negative, and rheumatoid factor (RF) was 15 IU/L. Serum antinuclear antibody titer was positive at 1 : 320, anti-SS-A antibody titer was positive at 1 : 256, but anti-SS-B antibody was negative. Other antibodies and immunological profile are shown in Table 1. Pleurocentesis was performed; on pleural fluid analysis, antinuclear antibody titer was 1 : 320 and anti-SS-A antibody titer was 1 : 256. Ophthalmological examination revealed a positive Schirmer test. Lip biopsy showed atrophy of the salivary gland and plasmacytic infiltration around the salivary gland ducts (Figure 2), consistent with SS. Ultimately, we diagnosed the patient with SS due to the presence of sicca symptoms. Corticosteroid treatment (prednisolone 40 mg/day) was initiated and produced a drastic decrease in the pleural effusions. Daily prednisolone dose was gradually reduced from 40 mg to 25 mg over three weeks. Pleural effusion was almost completely resolved by day 30. There is no evidence of recurrence thus far.

## 3. Discussion

In 1989 a European research group proposed classification criterion for SS; this was revised by an American-European consensus group in 2002 [9, 10]. It comprises two subjective criteria, ocular and oral symptoms, four objective criteria including ocular and oral signs, and histopathological and serological findings including antinuclear, anti-SS-A, or anti-SS-B antibodies. The diagnosis of SS requires at least four of the six criteria including histopathological or serological finding, or three of the four objective criteria. Our patient had ocular and oral symptoms and a positive Schirmer test. Furthermore, lip biopsy showed atrophy of the salivary gland with plasmacytic infiltration, and positive antinuclear and anti-SS-A antibodies were detected.

SS is classified into two types: primary and secondary, with the secondary form being complicated by other collagen disorders such as rheumatoid arthritis (RA), systemic lupus erythematosus (SLE), and scleroderma. In this case, the patient had no clinical signs of RA or scleroderma. There were some laboratory findings compatible with SLE, but only two criteria of SLE were met: serositis and high titer antinuclear antibody; thus we could not make the diagnosis of SLE. Eventually, a diagnosis of primary SS was reached.

SS is rarely accompanied by pleural effusion. Papathanasiou et al. [11] reported that pleural effusion was observed in no cases of primary SS and in 2 of 26 cases of secondary SS. There have been only six reports of primary SjS complicated by pleural effusion [3–8] (Table 1). Among them, pleural effusion was an initial manifestation only in two cases [6, 8]. Anti-SS-A and/or SS-B antibody was detected in the pleural effusion of these cases. Physicians should take notice to examine these antibodies in undiagnosed pleural effusion.

 Corticosteroid therapy, started at 30 or 40 mg/day of prednisolone if not complicated by interstitial pneumonitis, is a common treatment for SS [4]. A good response is expected, but recurrence is also reported during dose reduction [8]. There has been no evidence of recurrence in our patient so far, but careful followup is warranted.

 In conclusion, SS should be considered as one of the collagen diseases potentially presenting with pleuritis.

## Figures and Tables

**Table 1:** Autoantibody and immunological profile.

	Value	Normal value	Unit
Rheumatoid factor	15	0–10	IU/L
IgG	2693	870–1700	mg/dL
IgG4	35.4	4.8–105	mg/dL
IgA	525	110–410	mg/dL
IgM	194	33–190	mg/dL
Antinuclear antibody	X320	<40	
Homogenous	X320	—	
Speckled	X320	—	
Anti-ds-DNA antibody	10	<12	IU/mL
Anticardiolipin antibody (IgM)	1.2	<3.5	IU/mL
Anticardiolipin antibody (IgG)	8	<10	IU/mL
Lupus erythematosus test	Negative	—	
Lupus anticoagalant	1.05	<1.3	sec
Preneutralization	32.7	—	sec
Postneutralization	31.2	—	sec
Antiribonucleoprotein antibody	Negative	—	
Anti-Sm antibody	Negative	—	
Anti-Sjögren's syndrome-A antibody	X256	—	
Anti-Sjögren's syndrome-B antibody	Negative	—	
Proteinase-3 antineutrophil cytoplasmic antibody	<10	<10	EU
Myeloperoxidase antineutrophil cytoplasmic antibody	<10	<20	EU
Serum complement level	40.8	25–48	CH50/mL
C3	93	86–160	mg/dL
C4	19	17–45	mg/dL
Soluble interleukin 2 receptor	1550	145–519	IU/mL

**Table 2:** Literature review of primary Sjögren's syndrome complicated by pleural effusions [3–8].

Authors	Age	Gender	Chief symptoms	ANA		Anti-SS-A		Anti-SS-B	
				Serum	PE	Serum	PE	Serum	PE
Alvarez-Sala et al.	64	F	Chest pain	+	ND	−	ND	+	ND
Ogihara et al.	62	M	Fever	1 : 40	ND	1 : 4	1 : 4	1 : 8	1 : 8
Suzuki et al.	53	F	Cough	1 : 160	1 : 80	+	ND	+	ND
Kawamata et al.	70	M	Cough	1 : 1280	ND	+	+	−	−
Horita et al.	73	M	Dyspnea	1 : 320	ND	25.9 U/mL	22.3 U/mL	59.1 U/mL	76.4 U/mL
Teshigawara et al.	65	M	Cough, dyspnea	1 : 320	1 : 80	>500 U/mL	89.9 U/mL	49 U/mL	34.3 U/mL

ANA: antinuclear antibody, SS: Sjögren's syndrome, PE: pleural effusion, ND: not done.
